# “Queer people are excellent caregivers, but we’re stretched so very thin”: Psychosocial wellbeing and impacts of caregiving among LGBTQI cancer carers

**DOI:** 10.1186/s12885-023-11732-2

**Published:** 2024-01-05

**Authors:** Kimberley Allison, Rosalie Power, Jane M. Ussher, Janette Perz, the Out with Cancer Study Team, Alexandra Hawkey, Chloe Parton, Lucy Watson, Martha Hickey, Gary W. Dowsett, Antoinette Anazodo, Katherine Boydell, Jenni Bruce, Tenley Gilmore, Sam Ryan, Colin Ellis

**Affiliations:** Translational Health Research Institute, School of Medicine, Western Sydney University, Sydney, Australia

**Keywords:** Cancer carers, LGBTQI, Mixed methods, Qualitative, Psycho-social, Distress, Identity, Social support, Healthcare professionals

## Abstract

**Background:**

LGBTQI (lesbian, gay, bisexual, transgender, queer and/or intersex) communities are increasingly recognized as a vulnerable and high-risk population in oncology. LGBTQI cancer carers, including carers who are LGBTQI and other carers of LGBTQI people, experience many of the same stressors as LGBTQI patients but their support needs are often overlooked in the cancer literature.

**Method:**

This mixed-methods study examined distress and quality of life in LGBTQI cancer carers. Online surveys were completed by 129 carers and 31 carers took part in a one-to-one semi-structured interview. Analyses of variance (ANOVAs) tested for differences in psychosocial outcomes and carer experiences by gender, sexuality, age, carer relationship and carer/patient LGBTQI status. Reflexive thematic analysis of interviews and open-ended survey responses facilitated in-depth examination of subjective experiences.

**Results:**

42.6% of participants reported high or very high distress. Distress was significantly positively correlated with discrimination in cancer care, health impact, financial impact and lack of family support; it was negatively correlated with comfort in LGBTQI sexuality and gender identity, social support and quality of life. Four themes were identified in thematic analysis of qualitative data: (1) Identity on the sidelines: LGBTQI sexuality and gender pushed aside during cancer caregiving; (2) Fear of being shut-out: rejection and exclusion of LGBTQI cancer carers; (3) Lack of support for LGBTQI caregivers; and (4) Closer and stronger relationships due to a culture of mutual caregiving.

**Conclusions:**

LGBTQI cancer carers must contend with typical caregiving demands whilst also managing additional minority stressors, including discrimination, rejection from family, isolation from LGBTQI communities, and invisibility in healthcare and support services. Despite this, LGBTQI carers showed resilience in building their own mutually supportive networks to rally around the person with cancer, which were reported to ameliorate psychosocial vulnerabilities. Service providers need to recognize the needs of LGBTQI cancer carers through inclusive and reflective practices. This will facilitate trust and patient and carer sexuality and gender identity disclosure, with positive consequences for wellbeing and satisfaction with cancer care.

## Background

LGBTQI (lesbian, gay, bisexual, transgender, queer and/or intersex) communities are increasingly recognized as a vulnerable and high-risk population in oncology. They report worse psychosocial outcomes than their non-LGBTQI counterparts [1–3], and are invisible and underserved by healthcare systems [2, 4–6]. The American Society for Clinical Oncology highlighted that there is insufficient knowledge of the experiences, wellbeing, needs and potential interventions for LGBTQI populations to improve health outcomes [7]. This is particularly true of LGBTQI cancer carers, who experience many of the same stressors as LGBTQI patients [5], but have been almost entirely overlooked in the cancer caring literature.

Previous research has identified how cancer caregiving can impact upon many domains of life. Informal cancer carers experience poorer physical and mental health compared with the general population, disrupted intimate and social relationships, financial burden and vocational impacts [8–11]. There is evidence that 20-30% of cancer carers are at high risk for psychiatric morbidity [12], and in some studies, report significantly higher rates of distress than people with cancer [13, 14]. Greater cancer caregiving burden and poorer psychological wellbeing are reported by women [15–19], partner-carers [17, 18], younger carers [17, 18], those with low social support [16, 17], and those caring for a patient with advanced cancer [16, 17]. However, many carers report positive aspects of the caregiving experience, including treasuring their time with those they care for, relational enhancement and increased feelings of self-worth and esteem [9, 19]. Higher levels of carer self-efficacy and self-esteem are associated with increased wellbeing [20] and more active engagement in supporting the cancer patient [21].

Limited quantitative research on LGBTQI cancer carers has focused on breast cancer patient-carer dyads, primarily partners, reporting no differences in stress [22] and QOL [23] between the carers of sexual minority women and heterosexual women [24]. The strongest predictor of sexual minority carers’ distress levels was the patient/survivor’s disclosure of sexual orientation: carer distress was also lower if they reported larger friend and family support networks [24]. In qualitative studies, partner-carers of LGBTQI cancer patients have reported being excluded from care [25, 26], not being offered supportive services typically offered to heterosexual couples [27], feeling uncomfortable or othered when accessing resources and support groups intended for carers [28], and not being able to access partner-inclusive and/or LGBTQI-specific support groups [29, 30]. Partner-carers can also be invisible if the patient has not disclosed to healthcare professionals (HCPs) for fear of negative reactions, discrimination or maltreatment [5, 26–28], or if legal systems, HCPs or the patients’ families do not recognize their relationships [27, 31].

Additionally, legacies of discrimination, prejudice and exclusion, described as minority stressor*s* [32–34], mean that LGBTQI cancer carers may experience additional life stress and absence of family and community support [26], adding to their caregiving burden [31]. These stressors are known to contribute to poorer physical and mental health among members of minority groups [35] including LGBTQI carers [36], which may be compounded for those who are multiply marginalised through intersections of sexuality, gender diversity, age and cultural background [37, 38]. LGBTQI social support networks are diverse, with ‘chosen family’, including former partners, friends and other LGBTQI people, identified as key supports for sexual minority men and women with cancer [27, 29, 39]. However, these non-marital and non-biologically related carers are also often excluded from healthcare and medical decision-making [27].

There has been a call for research on LGBTQI cancer and cancer care to acknowledge the broad spectrum of gender and sexuality diversity within an intersectional framework [7, 40]. To date, the LGBTQI cancer carer literature largely focuses on same-gender partners of older cisgender (cis) women with breast cancer. Little is known about experiences of same-gender partners of cis women with other cancers, gay and bisexual male and transgender (trans, binary and non-binary) carers, carers with intersex variations, younger LGBQTI cancer carers, and non-partners (including LGBTQI carers of non-LGBTQI patients). This mixed-methods study addresses these gaps in the literature by exploring the psychosocial wellbeing, experiences and impacts of caregiving among LGBTQI cancer carers, across a range of sexuality and gender identities, age groups, cancer types and carer relationships. In this paper, LGBTQI cancer carers includes LGBTQI carers of both LGBTQI people with cancer and non-LGBTQI people with cancer. Our key research questions were:

1. Does distress, quality of life, and experiences and impacts of caregiving differ by gender, sexuality, age, carer relationship, and carer/patient LGBTQI status?
2. What factors are associated with distress and quality of life in LGBTQI cancer carers across a broad range of cancer types?

## Methodology

### Study design

This study was part of the *Out with Cancer Study*, a mixed-methods project exploring LGBTQI experiences of cancer and cancer care from the perspectives of LGBTQI people with cancer, carers and HCPs [1, 5, 26, 41–44]. This paper presents data from an online survey and semi-structured interviews with LGBTQI cancer carers. The project adopted an integrated knowledge translation (iKT) framework [45], with a 46-member stakeholder advisory group comprising LGBTQI cancer survivors and carers, HCPs, and representatives from LGBTQI health and cancer support organisations. The advisory group were involved in co-design and co-production at all stages of the project, including study design, data collection, analysis, and dissemination.

The research team and advisory group included people of different genders (cis-men and women, trans-men and women, and non-binary people), sexuality identities (lesbian, gay, bisexual, queer and straight) and ages (young adults to older adults). Most were White and lived in metropolitan or regional locations in Australia, UK or USA. Discussion between team members with different personal characteristics facilitated reflexivity [46], including evaluation of the ways our social positions impacted the research and interpretation of data. LGBTQI research team and advisory group members and those who had been cancer caregivers provided valued insights into the concerns of LGBTQI carers, to make meaning of participant data.

Western Sydney University’s Human Research Ethics Committee provided primary ethics approval for this study (ref. no. H12664), with secondary approval (ref. no. 2019/09) obtained from ACON, a health organisation specialising in LGBTQ health and a partner in this study.

### Participants and recruitment

Participants were eligible for this study if they (a) were at least 15 years old, (b) had acted as an informal carer for someone with cancer, and (c) they identified as LGBTQI. The study was advertised through cancer and LGBTQI community organisations, at in-person LGBTQI events and cancer support groups, and via social media (Facebook, Twitter, Instagram). Participants were also encouraged to share the survey link with eligible contacts. The survey was open from September 2019 until September 2021. At the end of the survey participants were invited to volunteer for an interview. Full details of participants and recruitment are described in detail elsewhere (see for e.g., [5, 26]). Based on statistical assumptions for the detection a medium *R*^2^ deviation from zero in outcome variables (psychological wellbeing and HRQOL) (*f*^2^ = 0.15) based upon a 0.9 power level at a significance level of 0.05, a minimum target sample of 140 carers was required.

### Quantitative measures

#### Distress

The ten-item Kessler Psychological Distress Scale (K10 )[47] asked participants to rate how frequently they experienced distressing feelings over the past 30 days using a five-point Likert scale (*none of the time* – *all of the time*). Total distress scores are computed by summing item responses (range 10-50) and, following Australian Bureau of Statistics guidelines [48], these are classified as low (10-15), moderate (16-21), high (22-29) or very high (30-50) distress. In this study, the K10 had excellent internal consistency (Cronbach’s *α* = .93).

#### Quality of life

A single item asked participants to report their overall quality of life (QOL) over the past week on a seven-point Likert scale (*very poor* – *excellent*). This item was derived from the EORTC-QLQ-C30 [49], which is widely used in cancer research [50].

#### Minority stress

##### Comfort in being LGBTQI

After reviewing the literature on LGBTQI minority stress and identity measures [51–53], three items were selected to assess how much participants were comfortable being LGBTQI, kept careful control over who knew they were LGBTQI, and wished they were not LGBTQI (asked separately about LGBQ identities, trans identities, and intersex variations). Participants responded using a five-point Likert scale (*strongly disagree – strongly agree*). After reverse coding where appropriate, scores were totalled to produce an overall score (range 3-15, higher scores indicating greater comfort being LGBTQI). These items had acceptable internal consistency (Cronbach’s *α* = .76).

##### Discrimination

Two discrimination questions were adapted from a previous study of sexual minority breast cancer survivors [54]. Participants reported the extent to which they had experienced discrimination for being LGBQ, trans and/or having an intersex variation (asked in separate pathways) in their lives in general and as a cancer carer, using a four-point Likert scale (*not at all/a little/quite a bit/very much*).

#### Impacts and experiences of caregiving

##### Caregiver esteem

The seven-item caregiver esteem subscale of the Caregiver Reaction Assessment (CRA )[55] was used to assess positive experiences in caregiving. Participants rated their agreement with each item on a five-point Likert scale (*strongly disagree* – *strongly agree*), with responses summed within each subscale to produce an overall score (range 7-35). The scale had acceptable internal consistency (Cronbach’s *α* = .77).

##### Impact on relationships

Four items were developed to assess the impact of cancer caregiving on the carer’s relationships with (1) the patient, (2) family, excluding partners, (3) friends, and (4) colleagues. A fifth item assessed the impact of caregiving on the carer’s intimate and sexual relationships. Response options were adapted from the Illness Intrusiveness Ratings Scale (IIRS [56];), with participants rating each impact on a four-point Likert scale (*not at all* – *very much*).

##### LGBTQI impacts

Three items were developed to assess the impact of cancer caregiving on (1) feelings about being LGBTQI, (2) openness about being LGBTQI, and (3) involvement with LGBTQI communities. Responses were made using a four-point Likert scale (*not at all – very much*) derived from the IIRS [56]. These questions were asked separately for LGBQ sexuality, trans identity, and intersex variations. Cronbach’s alpha for the three items was .52.

##### Other impacts of Cancer caregiving

A series of four items was developed to assess the impact of cancer caregiving on the carer’s health, education, work, and finances. These were based on the format of the IIRS [56], and asked participants to rate each impact using a four-point Likert scale (*not at all* – *very much*). These items were supplemented by three subscales of the CRA [55] measuring the impact of caregiving on carers’ finances (3 items, range 3-15, Cronbach’s *α* = .77), daily life/schedule (5 items, range 5-25, Cronbach’s *α* = .74), and health (4 items, range 4-20, Cronbach’s *α* = .66). Responses to these items used a five-point Likert scale (*strongly disagree* – *strongly agree*).

#### Social support

Participants’ current levels of social support were assessed using the social support subscale of the Health Literacy Questionnaire (HLQ) [57]. Participants used a five-point Likert scale (*strongly disagree* – *strongly agree*) to respond to five statements on whether they have support from others, with scores averaged to produce an overall social support score (range 1-5, higher scores indicate greater support). The scale had good internal consistency in this study (Cronbach’s *α* = .89). Participants were also asked to report who their primary support people were during the cancer caring experience. Finally, the subscale of the CRA assessing lack of family support (5 items, range 5-25, Cronbach’s *α* = .75 )[55] was also included in the survey, with participants rating their agreement to each item using a five-point Likert scale (*strongly disagree* – *strongly agree*).

### Qualitative data collection

The online carer survey included two open-ended questions asking, “*What was the most difficult aspect of being a carer for you?”* and “*What was the most rewarding aspect of being a carer for you?”* Other open-ended questions were included after key measures (e.g., the CRA, discrimination questions, the social support scale) asking, “*Is there anything you would like to tell us about this?”* Carers’ experiences were explored in greater depth through one-on-one semi-structured interviews of approximately 1 h each. The interviews, conducted via phone or videoconferencing software by trained members of the research team, explored experiences and the impact of caregiving, including interactions with HCPs and involvement in care, the impact of caregiving on LGBTQI identities and relationships, and support networks. Questions were tailored to participants’ experiences based on their survey responses and were phrased to elicit open-ended responses such as, “*Can you tell me about the support you received related to being a cancer carer, if any?”* and *“Can you tell me about your experiences of care provided by HCPs?”* All participants provided written informed consent for their participation.

### Data handling and analysis

#### Quantitative data

The final dataset comprised 129 surveys. Descriptive statistics (means and standard deviations for continuous variables; numbers and valid percentages for categorical items) were calculated for all measures. To facilitate between-groups analyses, gender was recoded into three categories (cis female, cis male, trans) based on participants’ self-reported gender (male, female, non-binary and other) and sex assigned at birth (male, female, something else). Sexuality was recoded into three categories (lesbian/gay/homosexual, bisexual, queer); two heterosexual trans participants were excluded from analyses of sexuality because of low numbers but were included in other analyses. While five participants indicated they were born with intersex variations, none completed questions specifically relating to intersex status. Age at survey completion was dichotomised, with participants classified as adolescents and young adults (AYAs, 15-39 years) or older adults (40+ years), using an internationally established definition of AYA [58]. Finally, participants were classified as partners or non-partners, and the carer/patient’s LGBTQI status was recoded into two categories: LGBTQI carers of LGBTQI patients, LGBTQI carers of cis heterosexual patients. Carers who were not LGBTQI were excluded from analyses of carer/patient LGBTQI status due to small sample size; however, these were included in other analyses. A series of one way analyses of variance (ANOVAs) tested for differences in psychosocial outcomes and caregiving experiences by between-subjects variables: gender (3 levels: cis female, cis male, trans), sexuality (3 levels: lesbian/gay, bisexual, queer), age (2 levels: AYA, older adult), carer relationship (2 levels: partner-carer, non-partner-carer) and carer/patient LGBTQI status (2 levels: LGBTQI carer for non-LGBTQI patient, both patient and carer LGBTQI). A Bonferroni correction was applied to control for the elevated rate of type I errors when performing multiple comparisons; an alpha cut-off of .01 (.05 divided by five families of comparisons) was used to indicate significance. Due to low participant numbers, comparisons between carers with intersex variations (*n* = 5) and those without (*n* = 124) were not possible. Bivariate correlational analyses were run to explore associations between study variables to identify factors associated with carer distress and QOL.

#### Qualitative analysis

Thirty-one carers volunteered to take part in interviews (19 cis women, 6 cis men and 6 trans participants). All interviews were audio-recorded, transcribed, checked for accuracy by reading the transcript while listening to the audio recording, and de-identified. Open-ended survey and interview data were analysed using reflexive thematic analysis, an appropriate method for collaborative and reflexive interpretation of patterns of meaning across the data [59]. This method acknowledges the research teams’ active role in knowledge production “at the intersection of data, analytic process and subjectivity” ([59], p. 594) facilitating the rich nuanced reading of the data. Through a collaborative process with the stakeholder advisory group, a subset of interviews and the open-ended survey data was read through line by line to identify first-order codes. Through collaborative and iterative discussion and decision making, codes with commonalities were organized into higher-order codes in a comprehensive coding framework. The interview data and open-ended survey responses were then imported into NVIVO and coded. Consistency in codes and coders was checked by a senior member of the research team. Data under each code were then read and summarised to facilitate the identification and development of themes, with the stakeholder advisory group again consulted on the interpretation and reporting of data. Different perspectives on the same data helped the team reflect on codes and develop themes. Themes were then refined through discussion, re-organized and when consensus was reached final themes were developed. In addition to team discussions, strategies to address research rigor included prolonged engagement with the subject matter, a detailed audit trail, and team reflexivity. Interview participants were assigned pseudonyms for reporting, with brief demographic and cancer details provided for longer quotes in text (and presented for shorter quotes in Table 4).

## Results

### Participant characteristics

Table 1 presents the demographic and cancer characteristics of the carers who completed the survey. Most respondents were living in Australia (70.5%), White (82.2%), cis women (62.0%), and all identified as LGBTQI (98.4% sexuality diverse, 17.8% trans and 3.9% born with intersex variations). The majority were over 40 years old (72.9%). More diversity was evident in regionality and cancer type (Table 1).

**Table 1:** Demographic Characteristics of Survey Participants

Demographic/ Cancer Characteristic	Carers		Patients cared for by carers	
	*N*	*M (SD),* range	*N*	*M (SD),* range
Age at time of study (years)	129	50.2 (17.2), 15-76	–	–
Age at diagnosis (years)	123	42.6 (16.7), 0-70	118	50.5 (15.2), 1-92
	*N*	*n* (%)	*N*	*n* (%)
Country	129		–	–
Australia		91 (70.5%)		
United States of America		14 (10.9%)		
United Kingdom		8 (6.2%)		
New Zealand		6 (4.7%)		
Canada		4 (3.1%)		
Other^a^		6 (4.7%)		
Gender	129		129	
Cis female		80 (62.0%)		88 (68.2%)
Cis male		26 (20.2%)		35 (27.1%)
Trans^b^		23 (17.8%)		4 (3.1%)
Multiple PWCs with different identities		–		2 (1.6%)
Sexuality	129		129	
Lesbian, gay or homosexual		95 (73.6%)		79 (61.2%)
Bisexual or pansexual		17 (13.2%)		5 (3.9%)
Queer		12 (9.3%)		5 (3.9%)
Straight or heterosexual		2 (1.6%)		33 (25.6%)
Different or multiple identities		3 (2.3%)		1 (0.8%)
Not sure		–		6 (4.7%)
Intersex variation	129		129	
Yes		5 (3.9%)		0
No		124 (96.1%)		124 (96.1%)
Prefer not to answer		0		0
Not sure		–		5 (3.0%)
Race/ethnicity	129		–	–
Caucasian		106 (82.2%)		
Asian		5 (3.9%)		
Australian Aboriginal, Torres Strait Islander or Maori		4 (3.1%)		
Mixed background		6 (4.7%)		
Other/unclear background^c^		8 (6.2%)		
Education	128		–	–
Less than secondary		7 (5.5%)		
Secondary		17 (13.3%)		
Some post-secondary		9 (7.0%)		
Post-secondary		95 (74.2%)		
Location	129		–	–
Urban		66 (51.2%)		
Regional		48 (37.2%)		
Rural or remote		15 (11.6%)		
Relationship to patient	129		–	–
Partner/ex-partner		84 (65.1%)		
Family		31 (24.0%)		
Friend		11 (8.5%)		
Different relationship		1 (0.8%)		
Multiple patients/relationships		2 (1.6%)		
Provided care during	125		–	–
Intervention for cancer risk		30 (24.0%)		
Diagnosis		96 (76.8%)		
Curative treatment		91 (72.8%)		
Survivorship/follow-up care		72 (57.6%)		
Palliative/end-of-life care		48 (38.4%)		
Care provided	125		–	–
Emotional		125 (100.0%)		
Practical		109 (87.2%)		
Informational		80 (64.0%)		
Physical		77 (59.7%)		
Financial		50 (38.8%)		
Spiritual		37 (28.7%)		
Other responsibilities	125		–	–
Household responsibilities		105 (84.0%)		
Work/volunteering		38 (30.4%)		
Other caregiving		22 (17.6%)		
Dependent children		16 (12.8%)		
Cancer diagnosis (first)	–	–	126	
Brain				9 (7.1%)
Breast				37 (29.4%)
Cervical				3 (2.4%)
Colorectal				8 (6.3%)
Head/neck				10 (7.9%)
Leukaemia				5 (4.0%)
Lung				7 (5.6%)
Lymphoma				6 (4.8%)
Ovarian				13 (10.3%)
Prostate				7 (5.6%)
Skin				3 (2.4%)
Uterine				4 (3.2%)
Other^d^				11 (8.7%)
Not sure or unknown				3 (2.4%)
Cancer stage	–	–	126	
Localised				53 (42.1%)
Regional				42 (33.3%)
Distant/metastatic				23 (18.3%)
N/A (e.g. blood cancer)				1 (0.8%)
Not sure or unclear				7 (5.6%)
Subsequent cancers^e^	–	–	126	
ecurrence				30 (23.3%)
New primary cancer				19 (14.7%)
Treatment status	–	–	126	
No treatment yet				5 (3.9%)
On active curative treatment				12 (9.4%)
On maintenance treatment				19 (15.0%)
In remission/completed treatment				35 (27.6%)
Receiving palliative care (no further active treatment)				2 (1.6%)
Deceased				51 (40.2%)
Not sure, unclear, or multiple				3 (2.4%)

^a^Belize (*n* = 2), Argentina, Lebanon, Germany, Uganda (*n* = 1 each)

^b^16 (12.4%) non-binary, 5 (3.9%) trans female, 2 (1.6%) trans male; patients: 2 (1.5%) non-binary, 1 (0.8%) trans female, 0 trans male, 3 (2.3%) different or multiple identities

^c^Hispanic/Latine, Jewish (*n* = 2 each), African, Native American (*n* = 1 each), not clearly described (*n* = 2)

^d^Bladder, liver, pancreatic (*n* = 2 each), kidney, mesothelioma, pseudo myxoma perotini, stomach, thymus (*n* = 1 each)

^e^Respondents could select multiple if applicable

### Psychosocial wellbeing

Means and standard deviations for the whole sample on all study measures are presented in Table 2. Of 94 participants who completed the K10, 35 (37.2%) reported low distress, 19 (20.2%) reported moderate distress, 25 (26.6%) reported high distress, and 15 (16.0%) reported very high distress. The mean distress score was 21.1 (SD 9.0, range 10-50). The mean QOL score was 4.7 (SD 1.6, range 1-7).

**Table 2:** Psychosocial Outcomes by Patient-Carer Relationship, and for Total Sample

				*M (SD)*		
Outcome	*df*	*F*	*p*	Partners (*n* = 81)	Other carers (*n* = 48)	Total sample
Distress	1,94	0.642	.425	20.5 (8.1)	22.1 (10.3)	21.1 (9.0)
Quality of life	1,96	1.346	.249	4.8 (1.6)	4.4 (1.7)	4.7 (1.6)
Comfort in LGBTQ+ identity	1,99	**9.825**	**.002**	12.8 (1.8)	11.1 (3.6)	12.2 (2.7)
Discrimination (gen life)	1100	0.978	.325	1.9 (0.7)	2.1 (1.0)	2.0 (0.8)
Discrimination (as carer)	1102	0.144	.705	1.3 (0.6)	1.3 (0.8)	1.3 (0.7)
LGBTQI+ impact	1102	0.896	.346	6.1 (2.5)	5.6 (1.9)	5.9 (2.3)
Social support	1106	1.614	.207	3.9 (1.0)	3.6 (0.9)	3.8 (0.9)
Lack of family support	1113	0.627	.430	11.8 (3.6)	12.4 (5.3)	12.0 (4.2)
Caregiver esteem	1113	**5.197**	**.025**	30.4 (3.3)	28.6 (5.0)	29.8 (4.0)
Impact on intimate/sexual relationships	1,87	**7.483**	**.008**	2.5 (1.1)	1.8 (1.1)	2.3 (1.2)
Financial impact	1113	0.155	.694	6.9 (3.1)	7.2 (2.7)	7.0 (3.0)
Schedule impact	1113	1.231	.270	17.2 (3.9)	16.3 (4.5)	16.9 (4.1)
Health impact	1113	0.388	.535	9.5 (2.8)	9.9 (3.8)	9.6 (3.1)

#### Differences in psychosocial wellbeing between groups

There were no significant differences in distress by gender, carer relationship, or carer/patient LGBTQI status. There were no significant differences in QOL by gender, sexuality, age, carer relationship, or carer/patient LGBTQI status.

#### Minority stress

##### Comfort in sexuality and gender identity

Most participants agreed that they were comfortable in their sexuality and gender identities (*n* = 88, 91.7% LGBQ participants; *n* = 11, 100.0% trans participants). Only 6 (6.3%) LGBQ participants and 1 (10.0%) trans participant wished they were not LGBQ or trans, indicating low levels of internalised prejudice. Thirty (31.3%) LGBQ participants and 5 (45.5%) trans participants reported keeping careful control over who knew they were LGBQ/trans, reflecting moderate levels of concealment motivation. Comfort in LGBQ or trans identity differed significantly by age and patient/carer LGBQ or trans status. Greater comfort was reported by older adults (*M* = 12.7, *SD* = 2.2) compared with AYAs (*M =* 10.4*, SD =* 3.4*;* F_*1,99*_ *= 14.448, p < .001*), by partner-carers (*M* = 12.8, *SD* = 1.8) compared with non-partner-carers (*M* = 11.1, *SD* = 3.6; *F*_*1,99*_ = 9.825, *p* = .002), and by LGBTQI carers of LGBTQI patients (*M =* 12.7, *SD* = 2.0) compared with LGBTQI carers of non-LGBTQI patients (*M =* 10.2, *SD* = 3.8; *F*_1,96_ = 16.975, *p* < .001)*.*

##### Discrimination

**Fig. 1:**
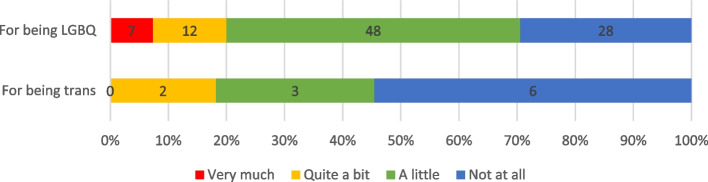
LGBTQI Carers’ Experiences of Discrimination in Life in General

**Fig. 2:**
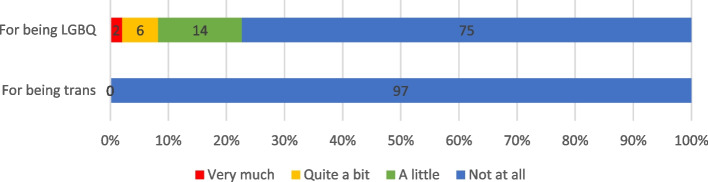
LGBTQI Carers’ Experiences of Discrimination in Cancer Care

Most participants (*n* = 71, 69.6%) reported experiencing discrimination in their lives in general for being LGBTQI, and 67 (70.5%) LGBQ people and 5 (45.5%) trans people had been discriminated against for their sexuality and trans identity, respectively (Fig. 1). A further 22 (21.2%) respondents had experienced discrimination in cancer care: all were LGBQ carers reporting sexuality-related discrimination (*n* = 22, 22.7%). No trans respondents reported anti-trans discrimination in cancer care (Fig. 2). There were no significant differences in experiences of discrimination by gender, sexuality, age, carer relationship, or carer/patient LGBTQI status.

#### Impacts of caregiving

##### Caregiver esteem

Respondents generally reported high caregiver esteem, with a mean score of 29.8 (SD 4.0, range 14-35) on the subscale of the Caregiver Reaction Assessment. Most participants agreed that they felt privileged to have cared for the patient (*n* = 99, 86.1%), really wanted to care for the patient (*n* = 104, 90.4%), and considered caregiving important to them (*n* = 113, 98.3%). Caregiver esteem did not differ significantly by gender, sexuality, age, or carer/patient LGBTQI status.

##### Relational impacts

Most participants reported relational impacts of caregiving, most commonly on the patient-carer relationship (*n* = 89, 79.5%), followed by relationships with families (excluding partners; *n* = 64, 59.3%), friends (*n* = 65, 60.7%) and colleagues (*n* = 38, 35.8%). Of 89 participants who were in intimate/sexual relationships during/since caregiving, 57 (64.0%) reported that caregiving had impacted upon these relationships: 15 (16.9%) reported a little impact, 25 (28.1%) quite a bit, and 17 (19.1%) very much impact. This impact was greater for partner-carers (*M* = 2.5, *SD* = 1.1) relative to non-partner carers (*M* = 1.8, *SD* = 1.1; *F*_*1,87*_ = 0.483, *p* = .008). There were no significant differences in relational impact by gender, sexuality, age, or patient/carer LGBTQI status.

##### Sexuality and gender identity

Most participants reported that cancer caregiving had impacted upon their sexuality and gender identities in some way. Thirty (28.8%) participants reported some impact on their feelings about being LGBQ (*n* = 24, 24.7%) or trans (*n* = 6, 54.5%); 64 (61.5%) reported some impact upon their openness about being LGBQ (*n* = 61, 62.9%) or trans (*n* = 4, 36.4%); and 62 (59.6%) reported some impact upon their involvement with LGBTQI communities (LGBQ *n* = 57, 58.8%; trans *n* = 6, 54.5%). Sexuality and gender identity impact did not differ significantly by gender, sexuality, age, carer relationship, or patient/carer LGBTQI status.

##### Other impacts of cancer caregiving

On the single-item measures, 62 (62.6%) respondents reported that cancer caregiving had impacted upon their health; 16 (16.0%) reported educational impact; 58 (58.0%) reported work/career impact; and 56 (56.0%) reported financial impact. Means for CRA subscales assessing financial, schedule and health impacts are presented in Table 2. The financial (*F*_2,108_ = 6.850, *p* = .002) impacts of caregiving differed significantly by carer sexuality. Financial impact was greater for bisexual participants (*M* = 9.7, *SD* = 2.6) than for queer (*M* = 7.6, *SD* = 3.4) and lesbian/gay participants (*M* = 6.5, *SD* = 2.8). There were no other significant differences in the impact of caregiving by gender, age, carer relationship, or patient/carer LGBTQI status.

#### Social support

Self-reported social support was high. The mean social support score from the HLQ was 3.8 (SD 0.9, range 1-5), and the mean score on the CRA subscale assessing lack of family support was 12.0 (SD 4.2, range 5-23). On the HLQ, most participants agreed that they had strong support from family and friends (71.1%), could get access to several people who understood and supported them (81.3%), and had plenty of people they could rely on if they needed help (66.3%). When asked to report their primary support people while they were caregiving, the most common responses were friends (*n* = 74, 69.2%), parents (*n* = 21, 19.6%), other family members (*n* = 47, 43.9%), partners (*n* = 31, 29.0%), and colleagues (*n* = 19, 17.8%). Only nine (8.4%) respondents indicated that they had no support people while caregiving. Social support did not otherwise differ significantly by gender, sexuality, carer relationship, or patient/carer LGBTQI status.

### Identifying factors associated with distress and QOL among LGBTQI Cancer caregivers

Table 3 presents bivariate correlations between study variables. Distress was significantly positively correlated with discrimination in cancer care, health impact, financial impact, and lack of family support. It was negatively correlated with comfort in sexuality or gender identity, social support and quality of life. Quality of life was positively correlated with comfort in sexuality or gender identity and social support, and negatively correlated with discrimination in cancer care, health impact, financial impact, and lack of family support.

**Table 3:** Bivariate Correlations between Study Variables

Variables	Social support	LGBTQ impact	Health impact	Schedule impact	Financial impact	Lack of family support	Caregiver esteem	Discrimination (as carer)	Discrimination (general life)	Comfort as LGBTQ	Quality of life	Distress
Distress	−.530^***^	.095	.357^***^	.189	.210^*^	.317^**^	−.007	.352^***^	.186	−.360^***^	−.666^***^	–
Quality of life	.362^***^	−.061	−.443^***^	−.186	−.301^**^	−.281^**^	.103	−.252^*^	−.129	.271^*^	–	
Comfort in being LGBTQI	.346^***^	.162	−.206^*^	−.034	−.113	−.095	.084	−.191	−.064	–		
Discrimination (general life)	−.143	.232^*^	.202^*^	.138	.263^**^	.283^**^	−.054	.443^***^	–			
Discrimination (as carer)	−.433^***^	.119	.205^*^	.177	.167	.444^***^	−.110	–				
Caregiver esteem	.205^*^	.050	−.199^*^	.015	−.265^**^	−.349^***^	–					
Lack of family support	−.538^***^	.137	.418^***^	.386^***^	.400^***^	–						
Financial impact	−.245^*^	.155	.521^***^	.348^***^	–							
Schedule impact	−.240^*^	.161	.580^***^	–								
Health impact	−.287^**^	.069	–									
LGBTQI impact	.016	–										
Social support	–											

*p < .05, **p < .01, ***p < .001

### Qualitative findings

Four themes were identified in the qualitative data: (1) Identity on the sidelines: LGBTQI sexuality and gender pushed aside during cancer caregiving; (2) Fear of being shut out: rejection and exclusion of LGBTQI cancer carers; (3) “It made me feel very alone”: lack of support for LGBTQI cancer carers; and (4) “Closer and stronger” relationships due to a culture of mutual caregiving. Participant demographics for the brief in-text quotes are included in the longer extracts presented in Table 4, identified with superscript letters after the in-text quote.

**Table 4:** Qualitative Themes and Quotes

**Identity on the sidelines: LGBTQI sexuality and gender pushed aside during cancer caregiving ** a. Well, at the time, I thought it wasn’t a priority for me. The focus is certainly on my partner and her wellbeing, her health. So whilst, you know, being a queer couple was out there straightaway, it also took a backseat. It was a very interesting dynamic because, regardless of whether or not the practitioner treated me in a different way, I always gotta make sure that her health and wellbeing was taken care of first and foremost. [Cameron, 38, non-binary, queer, caring for partner with breast cancer] b. That I felt that I had to pretend to be someone else was upsetting and stressful, like it was living in a disguise. I didn’t need the extra stress. [Cody, 38, non-binary queer, caring for grandmother with bowel cancer] **Fear of being shut out: rejection and exclusion of LGBTQI cancer carers** c. I am worried about this. My partner isn’t out with all her family and there is some avoidance about saying what she really wants. I know I’m in her Will but I’m not sure what it says. I feel like I have a world of pain coming if she does die. It will be unbearable. [Survey, 38, lesbian, caring for partner with brain cancer] d. We are a lesbian couple, not legally married. Thoughts about the legalities of not being legally married weighed on me, hoping that I never got ‘shut out’ of my partner’s care, [or prevented from] being with her, or being able to keep her in our home. [Survey, 56, lesbian, caring for partner with brain cancer] e. I had intense trauma as the gay partner of a cancer sufferer from her biological family, who wouldn’t recognize me or my role as her partner. [Survey, 59, cared for partner with ovarian cancer] f. My partner was closeted. She was also close to her religious family. Being her carer and navigating her family was incredibly difficult. [Survey, 59, queer, caring for partner with ovarian cancer] g. There is always an assumption that I’m her sister. Normally the first thing the nurses ask, “Is this your sister?” It was almost every time there was a change in shift until they got to know us, that’s nearly always the question, “Who’s your sister?” [Faye, 50, lesbian, caring for partner with leukemia] h. When they said to us, “Get your affairs in order”, we got the power of attorney stuff sorted. We may not have done that if my partner was confident that I could make the decisions for her. If we were a heterosexual couple, then that wouldn’t have been an issue for us. [Faye, 50, lesbian, caring for partner with leukemia] i. Being married I had the legal rights. Plus we had taken care of all legal paperwork years ago. [Survey, 62, gay, cared for partner with head/ neck cancer] j. I married my partner after she was diagnosed with a brain tumor and I believe the term wife gave me a lot more authority in a medical setting. [Survey, 61, lesbian, caring for wife with brain cancer] **“It made me feel very alone”: Lack of support for LGBTQI caregivers** k. Friends that had known us for years disappeared when he got sick. No one had the time to visit the funeral home. Not one word of encouragement. I quite frankly hate the gay community locally. They are only interested in gay pride and the bars. [Survey, 62, gay, cared for partner with head/ neck cancer] l. It’s devastating when you find that the people you thought were your supports become unreliable because of the relentless nature of caring, or because of their own grief. [Survey, 53, lesbian, caring for partner with ovarian cancer] m. I’ve pulled away from LGBTIQA+ specific events mostly due to being utterly exhausted still and not having the emotional energy to connect with others in this space. [Survey, 38, queer, non-binary, caring for partner with breast cancer] n. I have less emotional energy and time for others because I and my partner have so much to deal with. [Survey, 53, lesbian, caring for partner with breast cancer] o. Between COVID-19, working from home, my partner’s needs, my own mental health needs I am really feeling very alone and lost. [Survey, 39, queer, non-binary, cared for grandmother with brain cancer] p. I felt more isolated from the LGBTIQ+ community while I was a carer, I did not feel supported and also stopped interacting with this part of the community. [Survey, 38, non-binary queer, caring for grandmother with bowel cancer] q. I found a major lack of information and support for same-sex couples. [Survey, 47, lesbian, caring for partner with breast cancer] **“Closer” and “stronger” relationships due to a culture of mutual caregiving** r. It was a gift to be able to support her, provide a calm, caring environment. To take on the practical things she could focus on being with people. [Survey, 52, lesbian, caring for partner with lung cancer] s. Of course it’s hard work, but you have to care for the person you love [Survey, 29, bisexual man, caring for mother with ovarian cancer] t. Some of my friendships are much closer because they were excellent emotional support.” [Survey, 31, bisexual woman, caring for mother with breast cancer]	

#### Identity on the sidelines: LGBTQI sexuality and gender pushed aside during cancer caregiving

For several participants, being LGBTQI “took a backseat”^a^ while they were providing care to their partner, family member or friend with cancer. Participants, mostly of younger age, discussed delaying exploration and affirmation of their sexuality and gender identities, and grappling with decisions about disclosing identities with the person for whom they were caring. For example, Cedar, a 20-year-old, non-binary, bisexual person caring for their father who had brain cancer said, “I remember questioning my sexuality, like, ‘Hmm I don’t entirely feel straight’, but I’m already dealing with so much, I have no emotional energy left to deal with this. So, I just kind of ignored it”. Cody, a 38-year-old, non-binary, queer person caring for their grandmother who had terminal bowel cancer said that “as a carer, my identity as non-binary got pushed to the sidelines. I stopped taking hormones and presented as more feminine”. Cody explained their reasons for doing this were “two-fold”:

One, I did not want it [being non-binary] to affect my grandmother’s needs. Presenting as cis-gender made it easier to deal with medical professionals. The second part was it was just too difficult. To have my testosterone shot, I needed to go into the city. So, I wasn’t able to juggle my health needs and my medical needs with also balancing my grandmother.

Participants also discussed feeling conflicted about “coming out” while caregiving if the person they were caring for had terminal cancer. Zahra, an 18-year-old queer woman caring for her brother and mother both of whom had cancer said, “I don’t want to regret not telling my mum things like this”, but was concerned about the potential negative consequences. She explained, “I realized if I just say this one thing about myself [come out as queer], my mum will lose so many friends. We are Muslim, so many of her support groups will be affected because of me”. A 20-year-old lesbian survey participant who was caring for her father who had head and neck cancer said:

I never came out to my dad. I considered talking with him when he was definitely terminal, but I never did. I was scared of what he would say. I didn’t want to put extra stress on him in his final months.

These concerns caused some participants to feel they were “living in a disguise”^b^ while caregiving.

#### Fear of being shut out: rejection and exclusion of LGBTQI cancer carers

Anti-LGBTQI hostility and lack of legal protections for LGBTQI relationships meant that many participants felt “worried”^c^ they could be “shut out”^d^ from providing care for their partners with cancer and excluded from medical decision-making. Faye, a 50-year-old lesbian who was caring for her partner with leukaemia, said, “My partner and I were really concerned that if she became unwell and couldn’t make a decision—for example, if we needed to turn off her life support—that I wouldn’t be allowed to make that decision for her”. Fear of exclusion was often due to family members who “wouldn’t recognize me or my role”^e^ as a partner or carer. For example, Kai, a 59-year-old bisexual trans woman with intersex variations, said she had been “completely rejected by the family” after coming out as trans, requiring her to care for her partner “from afar”. Others said they had to conceal their relationships to provide care to their partners with cancer, because they were “closeted”^f^ due to anti-LGBTQI hostility from families. Navigating these circumstances was described as “incredibly difficult”.^f^ Lucinda, a 59-year-old queer woman who had cared for her partner with ovarian cancer told us that due to anti-LGBTQI hostility her 11-year relationship was erased by her partner’s family of origin:

For most of our relationship my partner was in the closet with her family because they were very anti-gay. When she was diagnosed with cancer they swooped in and basically said to me, ‘Off you go. We’ve got this now, piss off’, which was pretty tough. I was just like the best friend and the flat mate. And then she did die and – it all came out about our relationship, of course, you can’t really hide that stuff. They organized a full Catholic funeral for her where I wasn’t acknowledged or mentioned. I had to fight to be put on the gravestone. It was very traumatic. I’d been part of her family for 11 years and then basically I got wiped.

Many participants reported feeling invisible within and excluded by cancer services. Partner-carers said, “There was always an assumption”^g^ from HCPs that they were a sibling or a friend. A 72-year-old gay male survey participant who was caring for his husband with prostate cancer said that “despite being told we were married, nursing staff insisted on referring to me as my husband’s ‘friend’”. Non-partner, non-family caregivers also reported difficulties being recognized as legitimate carers. Scott, a 55-year-old trans man caring for his close friend with cancer, explained:

It was difficult for the medical profession. They did keep looking at me thinking, “Well, who exactly are you?” That this is odd. They do need to know who’s who and they do need to know whether I should be hearing all the confidential stuff. But I had no word for it [my caregiving role] and they had no category for me. It makes it exceptionally difficult.

A 60-year-old lesbian survey participant who, along with her wife, was caring for a friend with brain cancer said that they were treated “less respectfully” by HCPs than if they had been heterosexual, partner-carers.

To prevent potential exclusion, participants sought legal protections such as becoming appointed “power of attorney”^h^ or “being married”.^i^ This gave participants “a lot more authority in a medical setting”^j^ and capacity to advocate on behalf of their partners. A 62-year-old gay male survey participant whose partner had breast cancer said, “Being legally married has provided a level of certainty. We are a couple of two cultures and burial cultural considerations will be easier to enact now”.

#### “It made me feel very alone”: lack of support for LGBTQI cancer carers

Lack of recognition and exclusion of LGBTQI cancer carers from family of origin and within cancer services meant that many participants lacked adequate support in their roles as carers. A 60-year-old lesbian survey participant who had cared for her partner with breast cancer said, “Neither of our families offered much assistance. I had to ask people I didn’t know well if they could sit with my sleeping child so I could be beside my partner in hospital”. Other participants said, “Friends that had known us for years disappeared”,^k^ and that “people who we thought were our supports [became] unreliable because of the relentless nature of caring, or because of their own grief”.^l^ A 25-year-old survey participant caring for his partner with prostate cancer said, “I can’t seek support as I would need to come out and I can’t risk that”.

Lack of support from LGBTQI friends and communities was reported by some participants. A 38-year-old queer femme caring for their partner with an unknown cancer explained that multiple stressors impacted upon the capacities of LGBTQI people to support each other in times of need:

Queers are excellent caregivers, but we’re also stretched so very thin – trauma, discrimination, employment, housing, violence, mental health issues... You add physical illness, chronic illness and cancer care and no matter how great we are, there just aren’t enough of us to take care of each other, particularly those of us with no bio family and less money/security.

Although several participants said they intentionally “pulled away”^m^ from the LGBTQI community while caregiving, others said they felt unwelcome and that LGBTQI spaces were inaccessible for them and the person they were caring for. A 53-year-old lesbian survey participant caring for her partner with ovarian cancer commented:

It's difficult to engage with the wider queer community, just as it is with the rest of the world, when your partner is in a wheelchair, bald from chemo, bloated from steroids and exhausted all the time. No-one can relate.

These feelings of community estrangement combined with “having so much to deal with”^n^ meant that many participants felt “very alone”^o^ and “isolated”^p^ when caregiving.

For many participants, caregiving was marred by “a major lack of information and support”^q^ and feelings of exclusion from cancer caregiver support groups due to fears of hostility from other attendees. A survey respondent who was caring for their husband with leukaemia said, “I didn’t feel that I could go to support groups because I didn’t think I would be welcome there if I was honest about myself” (47, queer, non-binary, intersex person). Being “different” from other carers could also lead to feelings of exclusion. Leslie, a 32-year-old, queer non-binary person caring for their partner with breast cancer said:

Being queer really made it even more traumatic than it would have been anyway. For me to go to a breast cancer care and support group meant walking into a room of men who were 20 years older than me, where I didn't identify with the age or the gender or any of the problems that they were experiencing. It made me feel very different.

Another participant, a 53-year-old lesbian survey participant caring for her partner with breast cancer, said that she had been “offered help” by a HCP to set up an LGBTQI cancer support group, but that “required more emotional energy” during a time when she was already under strain due to her caregiving responsibilities. Lack of support for LGBTQI caregivers was reported to exacerbate the physical and psychosocial impacts of caregiving, as a 50-year-old asexual woman caring for her father with prostate cancer commented, “Caring for sick people on a long-term basis without adequate support makes you sick”.

#### Closer and stronger relationships due to a culture of mutual caregiving

Many participants reported that cancer caregiving was a catalyst for strengthened relationships with the person for whom they were caring. Most participants said that being able to support their partners, family members or friends with cancer was “a gift”^r^ and that although it was “hard work … you have to care for the person you love”.^s^ Darla, a 76-year-old lesbian caring for her lover with ovarian cancer said, “I wanted to be there for her all the way”. Barry, a 56-year-old gay man who had cared for his partner with lung cancer said:

Cancer is life-changing and in our case, an affirming experience. We have proven our strength of commitment to each other by the way we have responded to this dreadful challenge. It has made us love each other and rely on each other even more than before.

Several participants talked about increased closeness with queer chosen family members, including Alice, a 48-year-old lesbian, caring for her former partner with breast cancer. Alice’s wife and son were also involved in caregiving. Alice said, “This queer sense of family really fucks with other people’s norms. They expect that there’s going to be drama and are surprised when they see that we’re just all part of each other’s lives”. She explained, “It was a very conscious decision. My ex was brought into our family in a different, sort of, more intimate way for that period. Because of that, we’re forever going to be close”.

Many participants, particularly older lesbians, described receiving support in their role as carer from chosen family, attributed to a culture of mutual caregiving in LGBTQI communities. Lucinda, a 59-year-old queer woman, who had cared for her partner with ovarian cancer, described this support structure as a pyramid: “You’ve got that sick person at the top and then the immediate caregivers underneath and then they need support and then those people supporting those people need support and on it goes”. A 76-year-old lesbian survey participant caring for her partner with ovarian cancer said:

Being part of a quite large lesbian community who rallied and supported us whenever we needed it was extremely beneficial in terms of our emotional wellbeing and added to my feeling of not doing this alone in a mutually supportive way.

The “excellent emotional support”^t^ and practical assistance provided by chosen family helped participants to meet the demands of caregiving in a context of limited formal support.

## Discussion

This study examined LGBTQI cancer carers’ psychological wellbeing, experiences and impacts of caregiving, across gender, age, diagnoses, patient-carer relationships and identities, thereby expanding the limited literature on LGBTQI cancer caregiving. Findings highlight how LGBTQI carers must contend with typical caregiving demands [9–11] whilst also managing additional minority stressors linked to their sexuality or gender identities, including discrimination, rejection from family, isolation from LGBTQI communities, and invisibility in healthcare and support services. Despite this, LGBTQI carers showed resilience in building their own mutually supportive networks rallying around the person with cancer, and this was reported to ameliorate psychosocial vulnerabilities.

Over 40% of LGBTQI cancer caregivers reported high or very high distress, similar levels to LGBTQI cancer survivors in the *Out with Cancer Study* [1]. These distress levels were approximately seven times higher than previously reported for Australian general caregivers (6.9%) [60] and comparable to [61] or higher than [62] previous studies of cancer carers; however, they were somewhat lower than a number of other previous studies of cancer carers [63–65]. LGBTQI carers’ QOL in this study was almost identical to that of LGBTQI cancer survivors [1] and EORTC cancer population reference data [50]. These findings echo previous studies reporting similar QOL levels between sexual minority and heterosexual carers for women with breast cancer [22, 23], suggesting that LGBTQI cancer carers do not necessarily experience poorer quality of life than non-LGBTQI counterparts. There is need for further research on QOL in LGBTQI carers using more complex measurement tools than the single item used in the present study,  to identify nuances in carers’ QOL.

LGBTQI carers in this study reported unique minority stressors compared to the non-LGBTQI cancer carer population, including discrimination in general life and while caregiving, internalized prejudice and discomfort being LGBQ or trans, and the impact of caregiving on sexuality and gender identities and LGBTQ community involvement. While these have been discussed in previous qualitative studies on LGBTQI cancer carers [5, 26–28], this study provides an indication of their prevalence, as well as qualitative data to illustrate the nature of these experiences. Over two-thirds of participants reported discrimination in their lives, creating a “legacy” of distress and fear [26]. This legacy was evident in qualitative reports of actual and anticipated discrimination, exclusion from and invisibility in healthcare systems, contributing to distress, isolation and concealment, or sidelining of LGBQ or trans identities. Furthermore, over one-fifth of carers in this study had experienced discrimination as a cancer carer, which qualitative data suggested primarily took the form of exclusion from medical and supportive care and from patients’ support networks, and experiences of a range of fears that were also experienced by LGBTQI people with cancer [6, 26, 27]. This study identified that these experiences of discrimination and discomfort with LGBQ or trans identities were significantly associated with distress and QOL and may contribute to poorer psychological wellbeing amongst LGBTQI carers.

Accessed levels of social support were high, comparable to cancer patients and survivors in the *Out with Cancer Study* [1] and higher than has been previously reported for non-LGBTQI cancer caregivers [66–68]. As social support was associated with psychological wellbeing, as reported in previous research [20, 24, 69], this suggests that the strong support networks of study participants may have buffered the negative impact of minority stressors and caregiving burdens. When compared with previous studies using the CRA [70–78], LGBTQI cancer carers in this study reported lower family support, echoed in qualitative accounts of carers’ exclusion from family support networks. Combined with qualitative data indicating that LGBQ and trans carers felt excluded from cancer support groups [11, 12, 31, 33, 34, 39], a key source of support for people with cancer and their carers [79], LGBTQI carers may be being denied key sources of support available to other carers.

While many participants reported that they were supported by LGBTQI communities and chosen families, reflecting cultures of mutual caregiving [80, 81], others noted that cumulative care needs strained queer communities’ capacity to provide care during cancer, or felt that some queer spaces were unwelcoming or inaccessible to people with cancer and their carers. This echoes concerns previously reported by lesbian carers that caregiving impeded access to and involvement with queer communities and compromised queer identities, particularly among those who had to relocate or move in with non-LGBTQI family to provide care [82]. Clinicians and support organisations cannot simply delegate responsibility for supporting LGBTQI patients and carers to already overburdened queer communities; instead, mainstream service providers must take initiative in ensuring LGBTQI caregivers are welcomed, included and their needs met in cancer care systems [83].

Between-group analyses indicated few statistically significant differences between sexuality and age groups. For example, while bisexual and AYA participants reported greater distress than other groups, consistent with disparities previously evidenced for LGBTQI patients/survivors [1], these differences were not statistically significant after controlling for multiple comparisons. However, bisexual participants reported greater financial impacts, while AYAs reported less comfort in their LGBTQI identities. This hints at potential broader disparities in caregiving experiences that may emerge in larger studies. For bisexual carers, this may reflect psychosocial disparities in the broader LGBTQI population [84], associated with experiences of discrimination, invisibility and erasure [85]. AYA accounts evidenced how caregiving could disrupt critical processes of exploring and articulating LGBQ and trans identities, requiring AYA carers to play “catch up” with identity work and building LGBTQI community connections, as reported by AYA LGBTQI people with cancer in the *Out with Cancer Study* [86]. Further research is needed to elucidate the specific nature of experiences and support needs of younger LGBTQI cancer carers.

Previously reported differences in wellbeing between female and male informal cancer carers [71, 87, 88] were not found in the present study. This may reflect a less rigid adherence to binary gender roles in LGBTQI communities, which has been implicated in explaining these differences among heterosexual caregivers [87]. Finally, the lack of significant differences between partner-carers and non-partner-carers (other than comfort in sexuality and gender identities and impact on intimate/sexual relationships) highlights the need to ensure support services are made available and appropriate to all cancer carers – particularly as LGBTQI people with cancer are often supported by chosen family, including non-partner, non-family carers or may be in relationships that are not formally recognized as partners [27, 29, 39].

## Limitations

There were some limitations to our study. Recruitment of carers was challenging which limited our sample size – in particular, low numbers of intersex participants and people from diverse cultural backgrounds – limited the statistical power of analyses to draw conclusions on and identify differences between LGBTQI subgroups. Further research is needed to elicit further detail about the experiences and needs of these subpopulations to inform cancer services. Interviewees were predominantly cis women, as is the case with previous research on cancer carers [87]. Hence, future qualitative research is needed to explore the experiences of cis gay and bisexual men and trans cancer carers. A further limitation is that we used unvalidated measures where validated measures developed for the general cancer population were not appropriate for LGBTQI communities. Future research should validate existing scales for the LGBTQI population.

## Conclusion

This is the largest study to date to examine distress and QOL in LGBTQI cancer carers across LGBTQI identities, ages, cancer types and patient-carer relationships, contributing foundational knowledge to the limited extant literature in the area. The incorporation of both quantitative and qualitative data provides insight into the prevalence of, and association between, experiences and the impact of caregiving, as well as more nuanced accounts of how these are experienced in LGBTQI communities. This study’s findings may be useful in both service planning and the development of resources, including clinician training and patient/carer information, which aim to improve the quality and appropriateness of cancer care for this population.

Our findings have implications for cancer care and support services. It is important that all service providers recognize LGBTQI cancer carers, avoiding assumptions that carers, or the people they care for, are cisgender, heterosexual and do not have intersex variations. Awareness of the needs of LGBTQI cancer carers, through adoption of inclusive and reflective practice s[5], will enable HCPs to facilitate patient and carer disclosure, with positive consequences for patient and carer wellbeing and satisfaction with cancer care [89]. This can be achieved through education and training for HCPs [43], ensuring HCPs are knowledgeable and informed about broader sociocultural contexts that shape the experiences of LGBTQI carers, including higher risks of distress and barriers to social support [5]. Cancer information and support, including carer peer support, need to be LGBTQI inclusive and cancer environments need to signal LGBTQI awareness and safety [44]. There is also a need to extend research and knowledge translation to this area to, acknowledge and meet the needs of this population, ensuring positive implications for LGBTQI cancer carer outcomes.

## Data Availability

The datasets used and/or analysed during the current study are available from the corresponding author on reasonable request.
